# Grapevine Subtilase Family: Update on New Sequences and Nomenclature Proposal

**DOI:** 10.3389/fpls.2017.00716

**Published:** 2017-05-08

**Authors:** Joana Figueiredo, Marisa Maia, Marta Sousa Silva, Andreia Figueiredo

**Affiliations:** ^1^Faculdade de Ciências, Biosystems and Integrative Sciences Institute, Universidade de LisboaLisboa, Portugal; ^2^Laboratório de FTICR e Espectrometria de Massa Estrutural, Faculdade de Ciências, Universidade de LisboaLisboa, Portugal; ^3^Faculdade de Ciências, Centro de Química e Bioquímica, Universidade de LisboaLisboa, Portugal

**Keywords:** subtilases, *Vitis vinifera*, *Plasmopara viticola*, immunity, gene expression

## Abstract

In grapevine, serine peptidases from the subtilase family were recently associated to *Plasmopara viticola* resistance. This family in grapevine, first characterized in 2014, was re-analyzed last year and 82 subtilase genes were identified. However, in November of 2016, the National Center for Biotechnology Information database (NCBI) made a new public release of the grapevine genome annotation based on new sequencing data and better prediction algorithms. As a consequence, some gene annotations and lengths changed. Here we present an update to the grapevine subtilase gene family sequences (SBT), namely sequence identifiers, bioinformatic predictions and recommend a nomenclature for the grapevine SBT genes. Our results show that grapevine subtilase gene family is now constituted by 87 subtilase genes encoding for 109 subtilase proteins and, despite the reported alterations, expression data on subtilases associated to grapevine resistance to *P. viticola* pathosystem did not suffer any alteration.

Serine peptidases are the major class of proteolytic enzymes in plants, divided into 14 families. They are involved in all aspects of the plant life cycle ranging from the mobilization of storage proteins during seed germination to the initiation of cell death and senescence programs. Subtilisin-like proteases (SBTs), the second largest family of serine peptidases, present a broad spectrum of biological functions and, in the last years, its importance and participation in plant defense against the most diverse pathogens has been increasingly emphasized (reviewed in van der Hoorn, 2008).

Recently, Figueiredo's group have identified and characterized the grapevine subtilase gene family based on phylogenetic analysis, gene and protein primary structure predictions (Figueiredo et al., 2016). Furthermore, due to increased evidence of subtilase participation in plant immunity, they have performed gene expression analysis in grapevine-*Plasmopara viticola* pathosystem and associated some subtilases with a possible role in grapevine resistance against this oomycete. For the study, the authors used various bioinformatics tools and showed that this family is composed by 82 subtilase genes coding for 97 subtilase proteins.

The grape genome was sequenced in 2007 (Jaillon et al., 2007) and gene annotation was predicted with computational tools by comparison with *Populus trichocarpa, Arabidopsis thaliana*, and *Oryza sativa* genomes. Since November 2016, the National Center for Biotechnology Information database (NCBI) has made public a new release of the grapevine genome annotation based on new sequencing data and better prediction algorithms (https://www.ncbi.nlm.nih.gov/genome/annotation_euk/Vitis_vinifera/102/). We observed that the majority of the subtilase genes/proteins described in Figueiredo et al. (2016) did not perfectly match with the new release (Supplementary Data 1), from sequence identifiers to sequence length and annotation. Also, this new release has into account the latest grapevine genome release (V2) (Vitulo et al., 2014) and also 256 RNAseq libraries from different grapevine genotypes, tissues and under different physiological and stress conditions. This improvement represented major changes for 11% of grapevine genes and minor changes for around 71%.

Considering that plant subtilases are gaining more and more relevance and the number of research groups working in this area has been increasing, especially in the last 5 years, we consider that it is important to update the grapevine subtilase information already published to enable researchers to access the most correct and viable information. Thus, we have performed a re-analysis of the grapevine subtilase gene family taking into account the latest grapevine sequence information available in NCBI (November 2016). The bioinformatic tools used in this new analysis were the same as those described by Figueiredo's group. Moreover, we have added also the Grape Genome Database identifiers (http://genomes.cribi.unipd.it/grape) for all the subtilase sequences presented. The new results (see Supplementary Data 2 which replaces Supplementary Data 3 of the article Figueiredo et al., 2016) show that the grapevine subtilase gene family is constituted by 87 subtilase genes encoding for 109 subtilase proteins. A new phylogenetical analysis allowed re-defining the SBT groups based on nucleotide sequence similarity. Five SBT groups are proposed (Supplementary Data 2) as the out-group shown in Figueiredo et al. (2016) disappears. Moreover we recommend a nomenclature for the SBT genes (Supplementary Data 2) based on sequence similarity with *Arabidopsis* SBT genes as proposed by Grimplet et al. (2014).

The subtilase genes maintain the same chromosomal distribution as reported previously, being chromosome 13 the one with the highest number of genes (14) followed by chromosomes 16 and 15 with 9 and 8 subtilase genes, respectively. Concerning the number of introns, around 25% of the grapevine subtilase genes are intronless and 9% present a high number of these non-coding regions. Grapevine subtilase proteins are predicted to present the same molecular weight and isoelectric point ranges as previously reported. Subtilases are characterized by a multidomain structure composed by a signal peptide, an inhibitor I9 domain (PF05922), a peptidase S8 domain (PF00082) and a protease-associated (PA) domain (PF02225), (Tripathi and Sowdhamini, 2006). This new analysis revealed that the domain duplication previously predicted in some grapevine subtilases was due to sequence incorrections, thus none of the described subtilases present domain duplication. Despite this, and as expected, all the 109 proteins present the peptidase S8 domain, 103 contain the inhibitor I9 domain and only 60 present the protease-associated (PA) domain. Not all of the proteins presented simultaneously the S8, I9 and PA domains, the same as been previously described by Figueiredo et al. (2016). Of the 109 subtilases, 3 also contain the additional fibronectin (Fn) III-like domain (PF06280), which it is required for the activation of some subtilases. Regarding the prediction of subcellular location of grapevine subtilases, as already described by the authors, 80% of the proteins are predicted to be secreted, 10% are in the mitochondrion and 9% in the chloroplast. However, in this new analysis, we have detected 1 subtilase with prediction of localization in the cytoplasm (XP_010662319.1).

Despite the changes observed in the bioinformatic predictions for grapevine subtilases, results presented on subtilase expression analysis by qPCR on the grapevine- *P. viticola* pathosystem did not suffer any alteration.

## Author contributions

JF, MS, and AF conceived and wrote the manuscript; MM critically reviewed the manuscript.

### Conflict of interest statement

The authors declare that the research was conducted in the absence of any commercial or financial relationships that could be construed as a potential conflict of interest.

## Supplementary material

Supplementary Data 1 **Example of two grapevine subtilase sequences that were altered after NCBI new release on grapevine genome annotation (November 2016). (A)** Alignment of the previously published XP_010658658.1 (1430aa) sequence and the new sequence release XP_019079584.1 (697aa) highlighting that domain duplication seen in XP_010658658.1 was not correct; **(B)** alignment of the previously published XP_010658508.1 (525aa) sequence and the new sequence release XP_010658508.2 (762aa), highlighting that around 200aa were missing on the beginning of the XP_010658508.1 sequence.

Supplementary Data 2 **General features of grapevine subtilases**. Column A-Grapevine SBT groups based on phylogenetical analysis; column B- Vvi nomenclature; Bioinformatic predictions based on NCBI identifiers: columns C-Gene locus accessions, D-Nucleotide accessions, E-protein accessions, F-sequence description; column G-CRIBI gene identifiers. Columns H to X- predictions of, chromosomal location, number of intron and exons, enzyme code, protein length, molecular weight, isoelectric point, domain, Gene Ontology terms and subcellular location, for the VviSBT proteins described at NCBI (November 2016).
