# Supplementary material for: Grapevine Subtilase Family: Update on New Sequences and Nomenclature Proposal
Source: Front Plant Sci. 2017 May 8;8:716. doi: 10.3389/fpls.2017.00716 (PMC5420576; doi:10.3389/fpls.2017.00716)
Supplement: Supplementary Data 1 — Example of two grapevine subtilase sequences that were altered after NCBI new release on grapevine genome annotation (November 2016). (A) Alignment of the previously published XP_010658658.1 (1430aa) sequence and the new sequence release XP_019079584.1 (697aa) highlighting that domain duplication seen in XP_010658658.1 was not correct; (B) alignment of the previously published XP_010658508.1 (525aa) sequence and the new sequence release XP_010658508.2 (762aa), highlighting that around 200aa were missing on the beginning of the XP_010658508.1 sequence. [file DataSheet1.pdf]

A

```
XP_010658508.1      ------------------------------------------------------------      0
XP_010658508.2      MYRCPSTTKRIAYHHLMARQKNSSLWFLLLSLICSLLSTHSTAAASEDDVRKEYIVYMGA      60

XP_010658508.1      ------------------------------------------------------------      0
XP_010658508.2      KPAGDFSASAIHTNMLEQVFGSGRASSSLVRSYKRSFNGFVAKLTEDEMQQMKGMDGVVS      120

XP_010658508.1      ------------------------------------------------------------      0
XP_010658508.2      VFPSEKKQLHTTRSWDFVGFPRQVKRTSFESDIIIGVLDGGIWPESDSFDDKGFGPPPRK      180

XP_010658508.1      ---------------------------------------------------------MAS      3
XP_010658508.2      WKGTCQGFSNFTCNNKIIGAKYYKSDRKFSPEDLQSPRDSDGHGTHTASTAAGGLVNMAS      240
                                                                         ***

XP_010658508.1      LMGFGLGTARGGVPSARIAVYKICWSDGCDDADILAAFDDAIADGVDIISYSLGNPPSRD      63
XP_010658508.2      LMGFGLGTARGGVPSARIAVYKICWSDGCDDADILAAFDDAIADGVDIISYSLGNPPSRD      300
                    ************************************************************

XP_010658508.1      YFKDTAAIGAFHAMKNGILTSTSAGNDGPRLVSVVNVAPWSLSVAASTIDRKFLTEVQLG      123
XP_010658508.2      YFKDTAAIGAFHAMKNGILTSTSAGNDGPRLVSVVNVAPWSLSVAASTIDRKFLTEVQLG      360
                    ************************************************************

XP_010658508.1      DKKVYKGFSINAFEPNGMYPLIYGGDAPNTRGGFRGNTSRFCEINSLNPNLVKGKIVLCI      183
XP_010658508.2      DKKVYKGFSINAFEPNGMYPLIYGGDAPNTRGGFRGNTSRFCEINSLNPNLVKGKIVLCI      420
                    ************************************************************

XP_010658508.1      GLGAGFKEAWSAFLAGAVGTVIVDGLRLPKDSSNIYPLPASRLSAGDGKRIAYYISSTSN      243
XP_010658508.2      GLGAGFKEAWSAFLAGAVGTVIVDGLRLPKDSSNIYPLPASRLSAGDGKRIAYYISSTSN      480
                    ************************************************************

XP_010658508.1      PTASILKSIEVKDTLAPYVPSFSSRGPNNITHDLLKPDLTAPGVHILAAWSPISPISQMS      303
XP_010658508.2      PTASILKSIEVKDTLAPYVPSFSSRGPNNITHDLLKPDLTAPGVHILAAWSPISPISQMS      540
                    ************************************************************

XP_010658508.1      GDNRVAQYNILSGTSMACPHATGAAAYIKSFHPTWSPAAIKSALMTTATPMSARKNPEAE      363
XP_010658508.2      GDNRVAQYNILSGTSMACPHATGAAAYIKSFHPTWSPAAIKSALMTTATPMSARKNPEAE      600
                    ************************************************************

XP_010658508.1      FAYGAGNIDPVRAVHPGLVYDADEIDFVNFLCGEGYSIQTLRKVTGDHSVCSKATNGAVW      423
XP_010658508.2      FAYGAGNIDPVRAVHPGLVYDADEIDFVNFLCGEGYSIQTLRKVTGDHSVCSKATNGAVW      660
                    ************************************************************

XP_010658508.1      DLNYPSFALSIPYKESIARTFKRSVTNVGLPVSTYKATVIGAPKGLKINVKPNILSFTSI      483
XP_010658508.2      DLNYPSFALSIPYKESIARTFKRSVTNVGLPVSTYKATVIGAPKGLKINVKPNILSFTSI      720
                    ************************************************************

XP_010658508.1      GQKLSFVLKVEGRIVKDMVSASLVWDDGLHKVRSPIIVYAVQ  525
XP_010658508.2      GQKLSFVLKVEGRIVKDMVSASLVWDDGLHKVRSPIIVYAVQ  762
                    ******************************************
```

B

```
XP_010658658.1      MYRCPSTTKRIAYHHLMARQKNSSLWFLLLSLICSLLSTHSTAAASEDDVRKEYIVYMGA      60
XP_019079584.1      ------------------------------------------------------------      0

XP_010658658.1      KPAGDFSASAIHTNMLEQVFGSGRASSSLVRSYKRSFNGFVAKLTEDEMQQMKGMDGVVS      120
XP_019079584.1      ------------------------------------------------------------      0

XP_010658658.1      VFPNEKKQLHTTRSWDFVGFPRQVKRTSFESDIIIGVLDTGIWPESDSFDDKGFGPPPRK      180
XP_019079584.1      ------------------------------------------------------------      0

XP_010658658.1      WKGTCHGFSNFTCNNKIIGAKYYRSDGEFGREDLRSPRDSLGHGTHTASTAAGGLVSMAS      240
XP_019079584.1      ------------------------------------------------------------      0

XP_010658658.1      LMGFGLGTARGGVPSARIAVYKICWSDGCHGADVLAAFDDAIADGVDIISISAGSSTPSN      300
XP_019079584.1      ------------------------------------------------------------      0

XP_010658658.1      YFEDPIAIGAFHAMKNGILTSTSAGNEGPRFISITNFSPWSLSVAASTIDRKFFTKVKLG      360
XP_019079584.1      ------------------------------------------------------------      0

XP_010658658.1      DSKVYKGFSINTFELNDMYPLIYGGDAPNTRGGFRGNTSRFCKIKSLNPNLVKGKIVFCD      420
XP_019079584.1      ------------------------------------------------------------      0

XP_010658658.1      GKGGGKAAFLAGAIGTLMVDKLPKGFSSSFPLPASRLSVGDGRRIAHYINSTSDPTASIL      480
XP_019079584.1      ------------------------------------------------------------      0

XP_010658658.1      KSIEVNDTLAPYVPPFSSRGPNPITHDLLKPDLTSPGVHIVAAWSPISPISDVKGDNRVA      540
XP_019079584.1      ------------------------------------------------------------      0

XP_010658658.1      QYNIITGTSMACPHATGAAAYINAKKNPQVEFAYGAGNIDPVKAVHPGLVYDANEIDFVN      600
XP_019079584.1      ------------------------------------------------------------      0

XP_010658658.1      FLCGQGYTAKALRQVTGDHSVCSKATNGTVWNLNYPSFALSTFNKESIVGTFNRSVTNVG      660
XP_019079584.1      ------------------------------------------------------------      0

XP_010658658.1      LAVSTYKATIIGAPKGLKIKVKPNILSFTSIGQKQSFVLKVEGRIVEDIVSTSLVWDNGV      720
XP_019079584.1      ------------------------------------------------------------      0

XP_010658658.1      HQEYIVYMGDLPKGDISASTLHTNMLQQVFGSRASEYLLHSYQRSFNGFVAKLTMEEKKK      780
XP_019079584.1      -------MGDLPKGDISASTLHTNMLQQVFGSRASEYLLHSYQRSFNGFVAKLTMEEKKK      53
                           *****************************************************

XP_010658658.1      LSGIEGVVSVFPNGKKQLHTTRSWDFMGFPQKVKRTTTESDIIIGMLDTGIWPESASFSD      840
XP_019079584.1      LSGIEGVVSVFPNGKKQLHTTRSWDFMGFPQKVKRTTTESDIIIGMLDTGIWPESASFSD      113
                    ************************************************************

XP_010658658.1      EGFGPQPSKWKGTCQTSSNFTCNNKIIGARYYRTDGKLGPTDIKSPRDSLGHGTHTASTA      900
XP_019079584.1      EGFGPQPSKWKGTCQTSSNFTCNNKIIGARYYRTDGKLGPTDIKSPRDSLGHGTHTASTA      173
                    ************************************************************

XP_010658658.1      AGRMVRGASLLGLGSGAARGGVPSARIAVYKICWHDGCPDADILAAFDDAIADGVDIISL      960
XP_019079584.1      AGRMVRGASLLGLGSGAARGGVPSARIAVYKICWHDGCPDADILAAFDDAIADGVDIISL      233
                    ************************************************************

XP_010658658.1      SVGGYDPYDYFEDSIAIGAFHSMKNGILTSNSAGNTGPDPATITNFSPWSLSVAASTIDR      1020
XP_019079584.1      SVGGYDPYDYFEDSIAIGAFHSMKNGILTSNSAGNTGPDPATITNFSPWSLSVAASTIDR      293
                    ************************************************************

XP_010658658.1      KFVTKVKLGNNKVYEGVSVNTFEMDDMYPIIYGGDAPNTTGGYDSSYSRYCYEDSLDKSL      1080
XP_019079584.1      KFVTKVKLGNNKVYEGVSVNTFEMDDMYPIIYGGDAPNTTGGYDSSYSRYCYEDSLDKSL      353
                    ************************************************************

XP_010658658.1      VDGKIVLCDWLTSGKAAIAAGAVGTVMQDGGYSDSAYIYALPASYLDPRDGGKVHHYLNS      1140
XP_019079584.1      VDGKIVLCDWLTSGKAAIAAGAVGTVMQDGGYSDSAYIYALPASYLDPRDGGKVHHYLNS      413
                    ************************************************************

XP_010658658.1      TRYFCINSKPMAIIQKSVEVKDELAPFVVSFSSRGPNPITSDILKPDLTAPGVDILAAWT      1200
XP_019079584.1      T------SKPMAIIQKSVEVKDELAPFVVSFSSRGPNPITSDILKPDLTAPGVDILAAWT      467
                    *      *****************************************************

XP_010658658.1      EASSVTGKEGDTRVVPYSIISGTSMSCPHASAAAAYIKSFHPTWSPAAIKSALMTTAARM      1260
XP_019079584.1      EASSVTGKEGDTRVVPYSIISGTSMSCPHASAAAAYIKSFHPTWSPAAIKSALMTTAARM      527
                    ************************************************************

XP_010658658.1      SVKTNTDMEFAYGAGHIDPVKAVHPGLIYDAGEANYVNFLCGQGYSTKHLRLITGDKSTC      1320
XP_019079584.1      SVKTNTDMEFAYGAGHIDPVKAVHPGLIYDAGEANYVNFLCGQGYSTKHLRLITGDKSTC      587
                    ************************************************************

XP_010658658.1      SATMNGTVWDLNYPSFTISTKSGVTVTRIFTRTVTNVGSAVSTYKAILAVPSGLSVKVEP      1380
XP_019079584.1      SATMNGTVWDLNYPSFTISTKSGVTVTRIFTRTVTNVGSAVSTYKAILAVPSGLSVKVEP      647
                    ************************************************************

XP_010658658.1      SVLSFKSLGQKKTFTMTVGTAVDKGVISGSLVWDDGIHQVRSPIVAFVSS   1430
XP_019079584.1      SVLSFKSLGQKKTFTMTVGTAVDKGVISGSLVWDDGIHQVRSPIVAFVSS   697
                    **************************************************
```
